# Inhibition of autophagy prevents irradiation-induced neural stem and progenitor cell death in the juvenile mouse brain

**DOI:** 10.1038/cddis.2017.120

**Published:** 2017-03-23

**Authors:** Yafeng Wang, Kai Zhou, Tao Li, Yiran Xu, Cuicui Xie, Yanyan Sun, Yaodong Zhang, Juan Rodriguez, Klas Blomgren, Changlian Zhu

**Affiliations:** 1 Center for Brain Repair and Rehabilitation, Institute of Neuroscience and Physiology, Sahlgrenska Academy, University of Gothenburg, Gothenburg, Sweden; 2 Department of Pediatrics, Zhengzhou Children's Hospital, Zhengzhou, China; 3 Karolinska Institutet, Department of Women's and Children's Health, Karolinska University Hospital Q2:07, Stockholm, Sweden; 4 Henan Key Laboratory of Child Brain Injury, Third Affiliated Hospital of Zhengzhou University, Zhengzhou 450052, China; 5 Department of Pediatric Oncology, Karolinska University Hospital, Stockholm, Sweden

## Abstract

Radiotherapy is an effective tool in the treatment of malignant brain tumors. However, damage to brain stem and progenitor cells constitutes a major problem and is associated with long-term side effects. Autophagy has been shown to be involved in cell death, and the purpose of this study was to evaluate the effect of autophagy inhibition on neural stem and progenitor cell death in the juvenile brain. Ten-day-old selective Atg7 knockout (KO) mice and wild-type (WT) littermates were subjected to a single 6Gy dose of whole-brain irradiation. Cell death and proliferation as well as microglia activation and inflammation were evaluated in the dentate gyrus of the hippocampus and in the cerebellum at 6 h after irradiation. We found that cell death was reduced in Atg7 KO compared with WT mice at 6 h after irradiation. The number of activated microglia increased significantly in both the dentate gyrus and the cerebellum of WT mice after irradiation, but the increase was lower in the Atg7 KO mice. The levels of proinflammatory cytokines and chemokines decreased, especially in the cerebellum, in the Atg7 KO group. These results suggest that autophagy might be a potential target for preventing radiotherapy-induced neural stem and progenitor cell death and its associated long-term side effects.

Radiotherapy is one of the most effective tools in the treatment of malignant tumors, and it is used not only with adult patients but also with children who suffer from primary or metastatic brain tumors and from central nervous system (CNS) involvement of leukemia or lymphoma.^1, 2^ Irradiation to the whole body, including the brain, is also included in some protocols before hematopoietic stem cell transplantation. Damage to normal brain tissue surrounding the tumor constitutes a major problem and is associated with adverse side effects, particularly in pediatric patients.^3, 4^ The young and developing brain represents a special concern because it is more sensitive to irradiation than the adult brain.^5, 6, 7^ Cognitive impairments, secondary malignancies, and perturbed growth and puberty are some of the long-term effects after radiotherapy,^2, 8^ and irradiation-induced depletion of neural progenitor cells and stem cells appears to be long lasting, even after a single moderate dose of radiation.^9, 10, 11^ Thus, preventing irradiation-induced neural progenitor cell and stem cell death is an urgent issue that needs to be resolved so as to increase the clinical efficacy of radiotherapy.

Radiation damage can be induced either directly to the DNA or indirectly by the generation of reactive free radicals. DNA damage that escapes the cellular repair machinery can trigger a variety of cellular responses, including cell cycle arrest and cell death by mitotic catastrophe, necrosis, or apoptosis.^12, 13^ Mature neurons are considered to be in a permanent state of growth arrest, whereas stem and progenitor cells, as well as tumor cells, have high proliferative capacity and are therefore highly vulnerable to irradiation. Cell demise after irradiation, particularly in the developing brain, is to a large extent dependent on apoptosis-related mechanisms.^12, 13, 14^ Autophagy is essential for survival, differentiation, development, and homeostasis;^15, 16^ however, inappropriate activation of autophagy can be directly involved in mediating cell death or in triggering the initiation of apoptotic or necrotic cell death.^17, 18^ Induction of autophagy has been observed after irradiation in glioma cells,^19^ but whether induction of autophagy contributes to neuronal stem and progenitor cell death or protects against it is unclear.

The aim of this study was to investigate the effect of selective inhibition of autophagy on neuronal stem and progenitor cell death in the dentate gyrus and cerebellum in juvenile mice after cerebral irradiation and to elucidate the potential molecular mechanisms behind any such effect. We used mice deficient in autophagy specifically under a nestin promoter, including neuronally committed cells, due to the tissue-specific deletion of the *Atg7* gene, which is involved in autophagy induction and autophagosome formation.^16, 18^ We found that autophagy has an important role in the execution of neuronal progenitor and stem cell death in the neurogenic regions after irradiation in the juvenile mouse brain.

## Results

### Neuronal Atg7 deficiency reduces neural stem and progenitor cell death in the dentate gyrus and cerebellum

**Figure 1:**
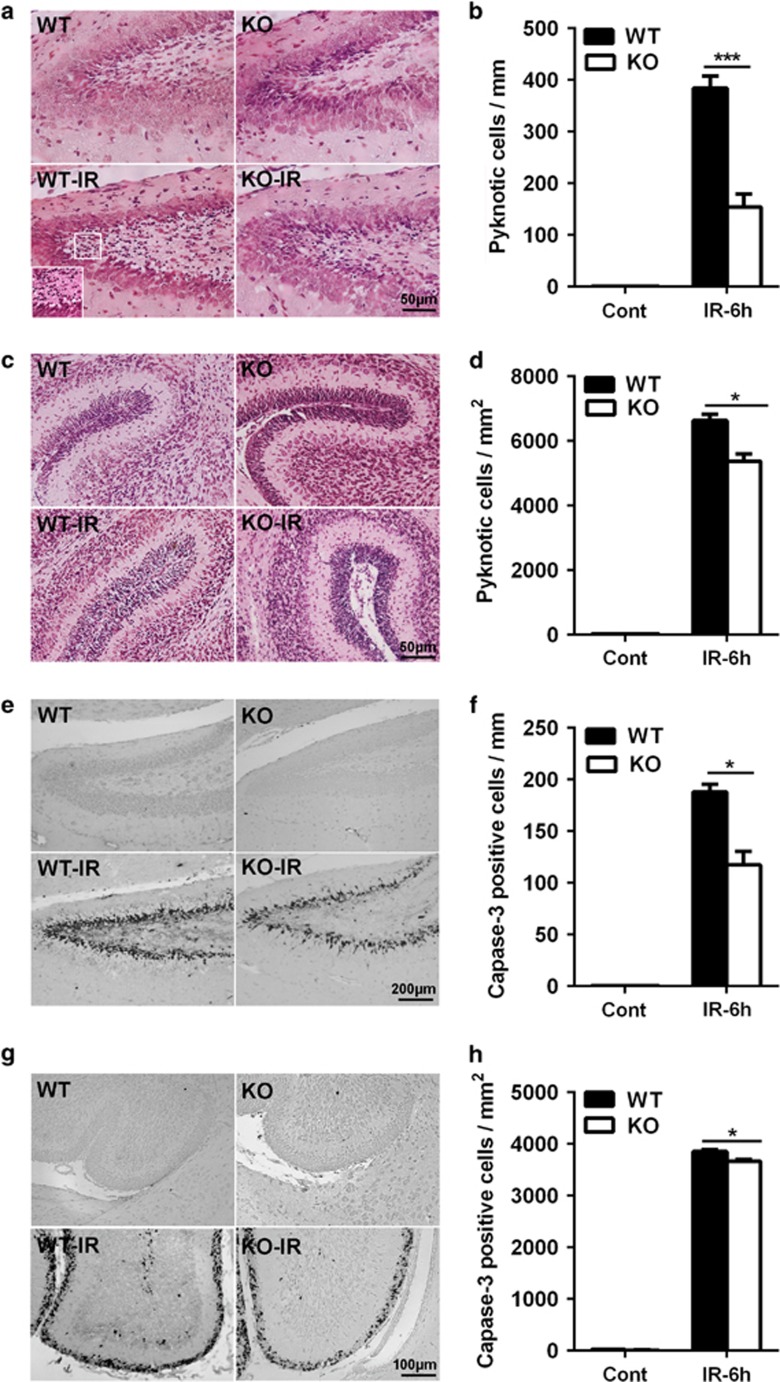
Neuronal Atg7 deficiency reduces neural stem and progenitor cell death in the dentate gyrus and cerebellum. (**a**) Representative hematoxylin and eosin (HE) staining in the dentate gyrus. (**b**) Quantification of pyknotic cells in the SGZ of the dentate gyrus. (**c**) Representative HE staining in a cerebellar lobule. (**d**) Bar graph showing the density of pyknotic cells in the EGL of the cerebellum. (**e**) Representative active caspase-3 staining in the dentate gyrus. (**f**) The density of active caspase-3-positive cells in the SGZ of the dentate gyrus. (**g**) Representative active caspase-3 staining in a cerebellar lobule. (**h**) The density of active caspase-3-positive cells in the EGL of the cerebellum; *n*=7 per group. *P<0.05 and ***P<0.001

Cell death was evaluated by counting pyknotic cells with hematoxylin and eosin (HE) staining (Figures 1a–d) and by counting active caspase-3-positive cells (Figures 1e–h) in the dentate gyrus (Figures 1a and e) and the cerebellum (Figures 1c and g). The pyknotic cells were mainly located in the subgranular zone (SGZ) of the dentate gyrus (Figure 1a), and the number of pyknotic cells increased at 6 h after irradiation. Neuronal Atg7 knockout (KO) reduced irradiation-induced pyknotic cell death by 60% in the dentate gyrus (384.4±22.6 pyknotic cells per mm in wild-type (WT) *versus* 154.3±24.8 pyknotic cells per mm in Atg7 KO mice after irradiation, *P*<0.001) (Figure 1b). The pyknotic cells in the cerebellum were mainly located in the external germinal layer (EGL) at 6 h after irradiation (Figure 1c), and the number of pyknotic cells in this region was reduced by 19% in the Atg7 KO group (6637.2±188.7 pyknotic cells per mm^2^ in WT *versus* 5376.4±228.6 cells per mm^2^ in Atg7 KO mice after irradiation, *P*=0.014) (Figure 1d). We further checked apoptotic cell death by staining for activated caspase-3, which is an established marker of apoptosis. There were very few cells immunopositive for active caspase-3 in the dentate gyrus (Figures 1e and f) and the cerebellum (Figures 1g and h) of the non-irradiated mice, but the number of active caspase-3-positive cells increased markedly after irradiation. Quantification showed a significant reduction in the number of active caspase-3-positive cells after irradiation in the Atg7 KO mice in the dentate gyrus (187.8±7.5 cells per mm in WT *versus* 117.4±12.8 cells per mm in Atg7 KO mice after irradiation, *P*=0.014) (Figure 1f) and the cerebellum (3854.7±36.2 cells per mm^2^ in WT *versus* 3670.3±29.2 cells per mm^2^ in Atg7 KO mice after irradiation, *P*=0.016) (Figure 1h).

### Neuronal Atg7 deficiency has no effect on neural stem/progenitor cell proliferation

**Figure 2:**
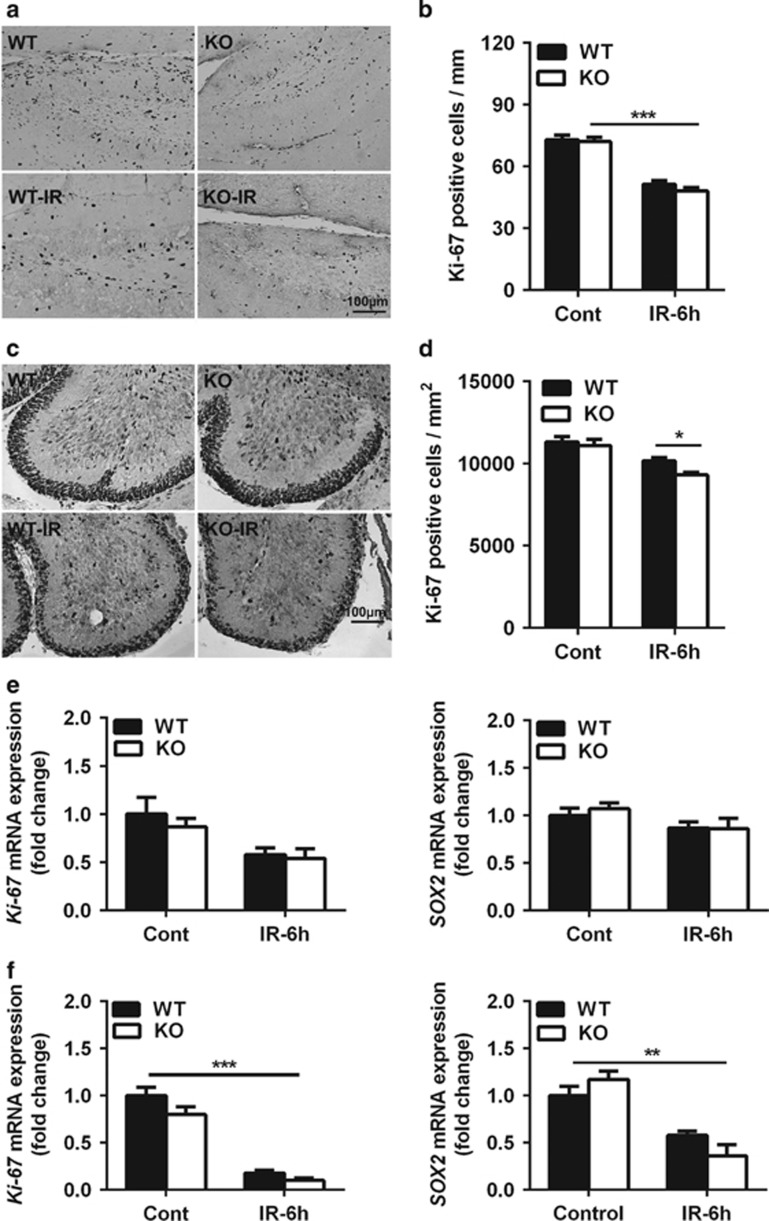
Neural stem and progenitor cell proliferation in the dentate gyrus and cerebellum. (**a**) Representative Ki-67 immunostaining in the dentate gyrus. (**b**) Quantification of Ki-67-positive cells in the SGZ of the dentate gyrus showed a significant decrease after irradiation. (**c**) Representative Ki-67 immunostaining in a cerebellar lobule. (**d**) Quantification of Ki-67-positive cells in the EGL of a cerebellar lobule. (**e**) The mRNA expression of *Ki-67* and *SOX2* in the hippocampus. (**f**) The mRNA expression of *Ki-67* and *SOX2* in the cerebellum decreased significantly after irradiation; *n*=7 per group for the immunostaining; *n*=5 per group for the quantitative PCR (qPCR) assays. *P<0.05 and **P<0.01

To determine the effect of neuronal Atg7 deficiency on neural stem and progenitor cell proliferation, Ki-67 immunostaining (Figures 2a–d) and mRNA expression (Figures 2e and f) were measured in both the dentate gyrus and the cerebellum. There was no significant difference in the number of Ki-67-labeled cells in the SGZ of the dentate gyrus (Figure 2b) or the EGL of the cerebellum (Figure 2d) between the non-irradiated Atg7 KO and WT mice. The number of Ki-67-positive cells decreased significantly at 6 h after irradiation in the SGZ of the dentate gyrus compared with the non-irradiated groups (*P*<0.001), but there was no significant difference between Atg7 KO and WT after irradiation (Figure 2b). The density of Ki-67-labeled cells decreased in the EGL of the cerebellum at 6 h after irradiation, and this effect was more pronounced in the Atg7 KO mice (9314.1±152.8 cells per mm^2^ in Atg7 KO *versus* 10 178.1±188.6 cells per mm^2^ in WT mice after irradiation, *P*=0.031) (Figure 2d). The expression of *Ki-67* mRNA decreased after irradiation in both the dentate gyrus (Figure 2e, left panel) and the cerebellum (Figure 2f, left panel), and the effect was more pronounced in the cerebellum, but there was no difference between Atg7 KO and WT (Figures 2e and f, left panel). The non-mitotic neural stem and progenitor cells, as indicated by *SOX2* mRNA expression, showed no difference between Atg7 KO and WT in the dentate gyrus, and there was no difference compared with controls at 6 h after irradiation (Figure 2e, right panel). The *SOX2* mRNA level in the whole cerebellum was decreased more than 50% after irradiation (Figures 2e and f, right panel).

### Neuronal Atg7 deficiency reduces irradiation-induced microglia activation

**Figure 3:**
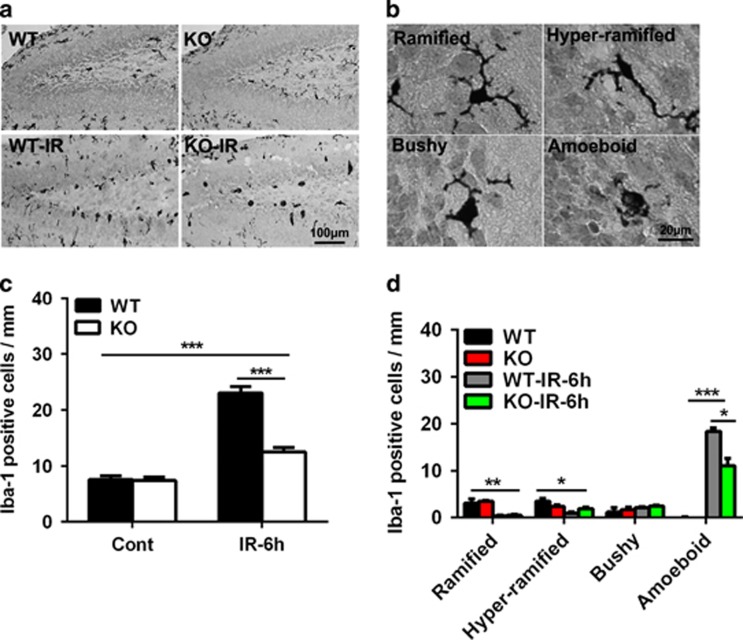
Neuronal Atg7 deficiency reduces microglia activation in the dentate gyrus. (**a**) Representative Iba-1 immunostaining in the dentate gyrus. (**b**) Representative morphology of Iba-1-positive cells indicating ramified (surveillance microglia), hyper-ramified (intermediate), and unramified (bushy or amoeboid) microglia. (**c**) Iba-1-positive cells were significantly increased in the SGZ of the dentate gyrus in both WT and KO mice after irradiation. (**d**) Quantification of Iba-1-positive cells according to morphology in the SGZ of the dentate gyrus at 6 h after irradiation; *n*=7 per group. *P<0.05, **P<0.01, and ***P<0.001

We and others previously showed that irradiation induces microglia activation and inflammation in the neurogenic regions^11, 20, 21, 22^ and that autophagy inhibition reduces cytokine and chemokine expression and microglia activation.^18^ Thus, we investigated the effect of neuronal Atg7 deficiency on some inflammatory markers and signaling pathways in the regulation of microglia activation. Microglia, as indicated by Iba-1 labeling, were scattered throughout the normal brain and became unramified or activated (bushy or amoeboid) under pathological conditions (Figures 3a and b). The number of microglia in the SGZ of the dentate gyrus was not different between Atg7 KO and WT mice in the non-irradiated controls, but the number increased markedly after irradiation (*P*<0.001) and Atg7 KO reduced the increase by 45.5% (23.1±1.1 microglia cells per mm in WT *versus* 12.6±1.4 microglia cells per mm in Atg7 KO mice after irradiation, *P*<0.001) (Figure 3c). The number of activated microglia with amoeboid morphology was increased markedly, and the number of ramified or hyper-ramified microglia was decreased significantly after irradiation (Figure 3d).

**Figure 4:**
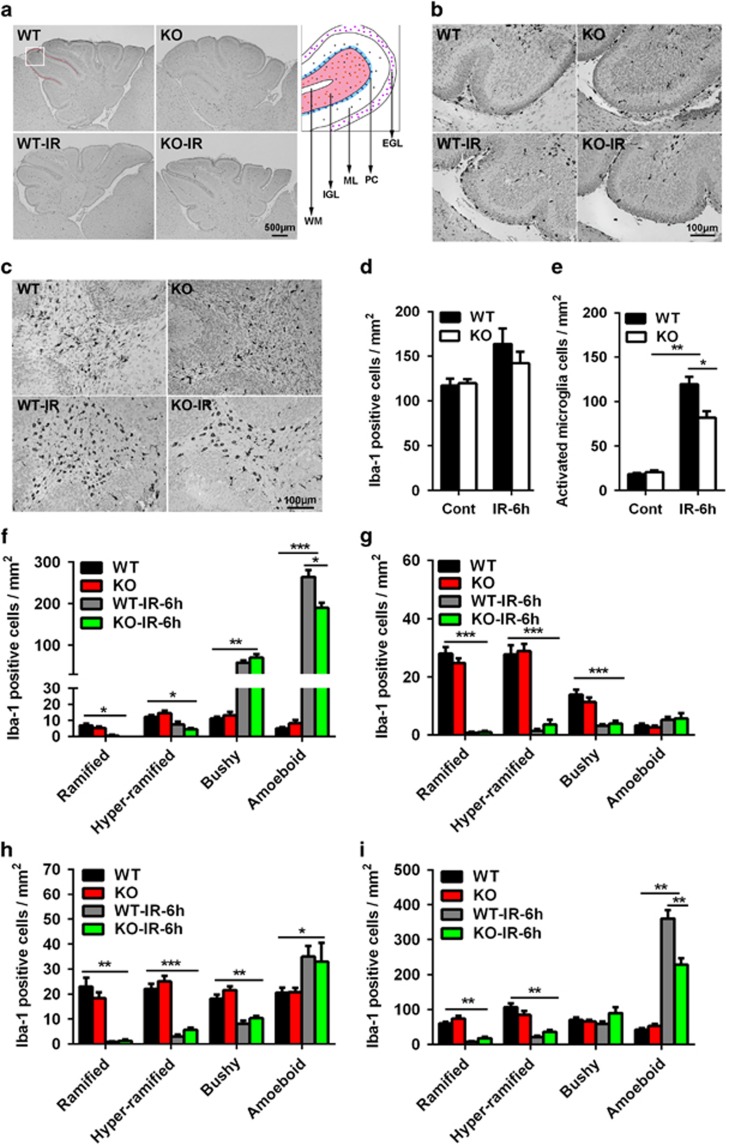
Neuronal Atg7 deficiency reduces microglia activation in the cerebellum. (**a**) Representative Iba-1 immunostaining in sagittal sections of the cerebellum. Each folia comprises distinct cellular layers: EGL; ML; Purkinje cell layer (PC), IGL, and WM. (**b**) Representative Iba-1 immunostaining in the EGL, ML, and IGL of a cerebellar lobule. (**c**) Representative Iba-1 immunostaining in the WM of a cerebellar lobule. (**d**) Quantification of total Iba-1-positive cells in the whole cerebellum. (**e**) Quantification of total activated microglia based on morphology in the whole cerebellum. (**f**) Quantification of Iba-1-positive cells according to morphology in the EGL of the whole cerebellum. (**g**) Quantification of Iba-1-positive cells according to morphology in the ML. (**h**) Quantification of Iba-1-positive cells in the cerebellar IGL. (**i**) Quantification of Iba-1-positive cells in the WM of the whole cerebellum; *n*=7 per group. *P<0.05, **P< 0.01, and ***P<0.001

The distribution of microglia in the cerebellum was uneven, and there were more microglia in the cerebellar white matter (WM) (Figures 4a–c). The density of microglia was slightly increased in the whole cerebellum after irradiation, but no significant difference was seen between Atg7 KO and WT in either the control or irradiated group (Figure 4d). However, the number of activated microglia, based on morphology, was increased significantly after irradiation but to a lesser degree in the Atg7 KO group (Figure 4e). Microglia with different morphology were counted separately in the cerebellar EGL (Figure 4f), molecular layer (ML) (Figure 4g), cerebellar internal granular layer (IGL) (Figure 4h), and cerebellar WM (Figure 4i). There were more activated microglia (bushy or amoeboid Iba-1-labeled cells) in the EGL and WM after irradiation, and the number was reduced in the Atg7 KO mice (Figures 4f and i).

### Effect of neuronal Atg7 deficiency on cytokine and chemokine expression

**Figure 5:**
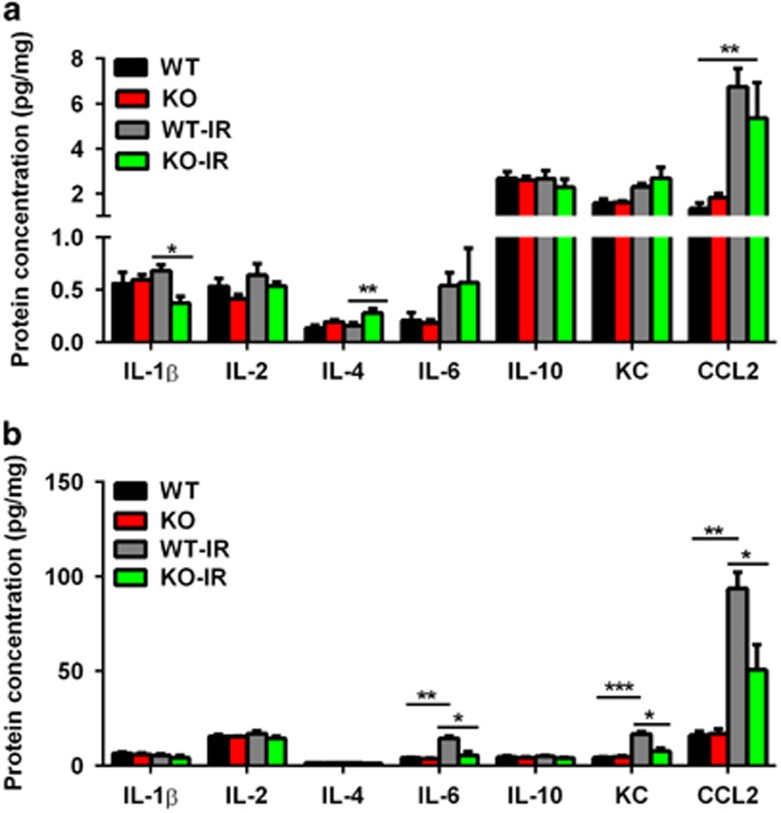
Cytokine and chemokine expression. (**a**) Luminex assay of cytokines (IL-1*β*, -2, -4, -6, and -10) and chemokines (KC and CCL2) in the cytosolic fraction of the hippocampus in Atg7 KO and WT mice. (**b**) Cytokine and chemokine expression in the cytosolic fraction of the cerebellum showed a decrease in IL-6, KC, and CCL2 in Atg7 KO mice compared with WT mice; *n*=7 per group. *P<0.05 and **P<0.01

Under pathological conditions, activated microglia produce proinflammatory cytokines and chemokines such as IL-1*β*, IL-2, IL-6, KC, and CCL2, which result in detrimental outcomes, and anti-inflammatory cytokines such as IL-4 and IL-10, which induce beneficial effects. The dysregulation of cytokines and chemokines is a central feature of neuroinflammation. In this study, we observed a decrease of IL-1*β* and an increase of IL-4 in the dentate gyrus in the Atg7 KO mice after irradiation. The level of CCL2 increased significantly after irradiation in both Atg7 KO and WT mice, but there was no difference in CCL2 between the two groups (Figure 5a). The basal levels of cytokines and chemokines were higher in the cerebellum than in the dentate gyrus (Figure 5b). The levels of IL-6, KC, and CCL-2 were increased significantly at 6 h after irradiation in the cerebellum in the WT mice compared with the controls, and Atg7 KO prevented the increase after irradiation.

### The mRNA expression of activated microglia-related genes

**Figure 6:**
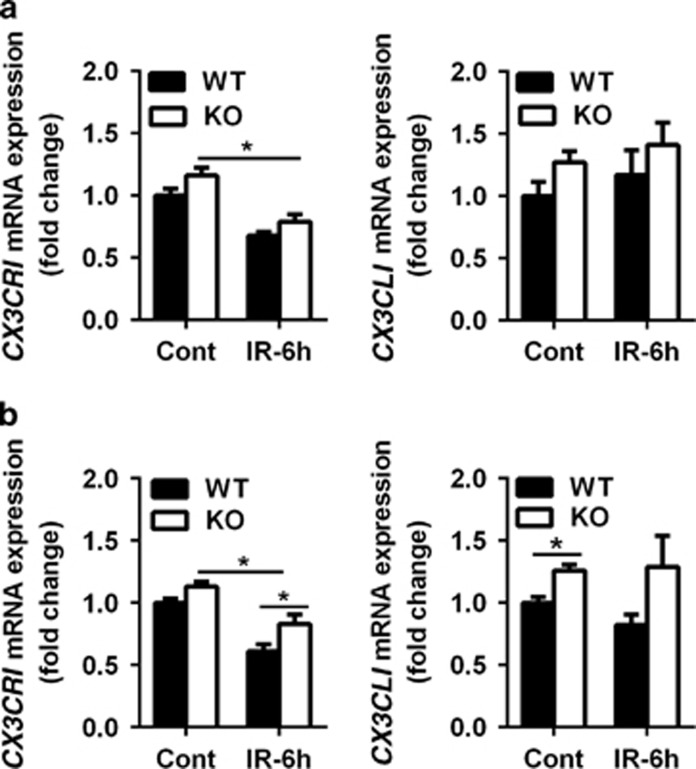
The mRNA expression of activated microglia-related genes. (**a**) Bar graphs showing mRNA expression of *CX3CR1* and *CX3CL1* in the dentate gyrus at 6 h after irradiation. (**b**) Bar graphs showing mRNA expression of *CX3CR1* and *CX3CL1* in the cerebellum at 6 h after irradiation; *n*=5 per group. *P<0.05

The *CX3CR1* and *CX3CL1* (fractalkine receptor and fractalkine) mRNA expression was checked in the dentate gyrus (Figure 6a) and the cerebellum (Figure 6b), and we found a significant reduction in *CX3CR1* mRNA expression in Atg7 KO and WT mice in both the dentate gyrus and the cerebellum after irradiation. The reduction was more pronounced in the cerebellum in the WT mice after irradiation (*P*=0.012), and there was no significant difference in the dentate gyrus between WT and Atg7 KO. The mRNA expression of *CX3CL1* was significantly higher in the Atg7 KO mice in the cerebellum under physiological conditions, but not in the dentate gyrus. Irradiation had no significant effect on *CX3CL1* mRNA expression in either the dentate gyrus or the cerebellum (Figures 6a and b).

## Discussion

Autophagy is a highly regulated process involving the bulk degradation of cytoplasmic macromolecules and organelles in mammalian cells via the lysosomal system, and it is induced under starvation conditions.^23, 24^ Selective KO of Atg7 in neuronally committed cells shows that basal levels of autophagy are required for normal neuron survival,^16, 25^ and autophagy is a protective mechanism in response to numerous stresses.^26, 27^ Overactivated autophagy induces autophagic cell death,^28^ and genetic inhibition of autophagy prevents hypoxia–ischemia-induced neuronal cell death in multiple brain regions.^18^ Autophagy has also been shown to be involved in irradiation-induced cell death in cancer cells,^29, 30^ and autophagy inhibitors can enhance radiosensitivity in gliomas;^31^ however, it is still unknown if inhibition of autophagy works to prevent irradiation-induced neural stem and progenitor cell death in the juvenile brain. In this study, we demonstrated that selective inhibition of autophagy in neurons reduced irradiation-induced neural stem and progenitor cell death in the dentate gyrus and the cerebellum in juvenile mice. These data suggest that autophagy inhibition could be used as a joint treatment with radiotherapy for malignant brain tumors, including high-grade gliomas.

We previously showed that clinically relevant doses of irradiation to developing rodent brains cause extensive damage to neural stem and progenitor cells and lead to a significant reduction of these cells,^3, 12, 13^ and even a single low dose of irradiation causes apoptotic cell death in different brain regions.^32^ We found in this work that neuron-specific KO of Atg7 reduces neural stem and progenitor cell death and caspase-dependent apoptosis after irradiation both in the dentate gyrus and the cerebellum. The functional relationship between apoptosis and autophagy is complex, and these two processes might be triggered by common upstream signals.^33, 34, 35^ Further research is needed to investigate the potential pathways and crosstalk between the two processes.

Autophagy has an important physiological role in the process of cell proliferation and differentiation.^15, 36^ Ki-67 is a nuclear protein that is associated with cellular proliferation and is present during all active phases of the cell cycle (G1, S, G2, and M), but is absent from resting cells, and therefore it has been widely used as a cellular marker of proliferation. Our counts of Ki-67-labeled cells and measurements of *Ki-67* mRNA expression showed that selective neuronal Atg7 deficiency has no obvious effect on neural stem and progenitor cell proliferation under either physiological or pathological conditions on postnatal day 10. This could be because Atg7 expression was not stopped until the cells were neuronally committed and/or because the effect of neuronal Atg7 deficiency on cell proliferation is developmental age-related and there is no significant effect during early postnatal life. Previous results showed that neuronal Atg7 deficiency yielded progressive behavioral deficits apparent from postnatal day 28.^16^ Irradiation-induced proliferating cell death might partly explain the reduction of Ki-67-labeled cells after irradiation, and the reduction was more pronounced with longer periods of recovery after irradiation.^9^ The sensitivity of different brain regions to irradiation varies,^37^ and our data suggest that the cerebellum is more sensitive to irradiation than the dentate gyrus in the juvenile mice as indicated by both *Ki-67* and *SOX2* mRNA expression. Our data also indicate that more neural stem and progenitor cells exist in the developing cerebellum, particularly in the cerebellar EGL, and this suggests that the cerebellum should be protected when radiotherapy is applied to children.

Microglia are the resident phagocytes of the CNS, and they are involved in the maintenance of brain homeostasis and immune defense. They have remarkable functional plasticity and the capacity to expand in response to injury and to acute or chronic diseases in the CNS.^38, 39^ Microglia become activated after injury, and this process involves morphological transformation and increased expression of pro- as well as anti-inflammatory chemokines and cytokines.^20, 40, 41, 42^ Irradiation causes oxidative stress and microglia activation, both of which can be toxic to other cells,^32^ and along with inflammation can induce cell death. Thus, inhibition of oxidative stress and microglia activation can provide neuroprotection during radiotherapy.^18, 43^ We found that microglia were activated as early as 6 h after irradiation and that the inhibition of autophagy reduces microglia proliferation and activation. Reduced microglia activation is accompanied by reduced levels of proinflammatory cytokines and chemokines and increased levels of anti-inflammatory cytokines, and this might account for the reduced neural stem and progenitor cell death in neuronal Atg7 KO mouse pups or *vice versa*. Autophagy has important roles in the regulation of microglia activation and inflammation,^44, 45^ and the interaction between autophagy and microglia activation needs to be investigated further.^45, 46^

The CX3CL1-CX3CR1 signaling pathway has been shown to have an important role in the regulation of microglia activation.^47^ It has been reported that CX3CL1 deficiency worsens the behavioral impairment after ischemic brain injury, and administration of exogenous CX3CL1 reduces brain injury and neurologic deficits.^48^ Other studies have found that lack or deficiency of CX3CR1 reduces brain injury and inflammation in a mouse model of cerebral ischemia^49^ and that CX3CL1-CX3CR1-mediated microglia activation has a detrimental role in ischemic mice.^50^ In this study, we found an increase in CX3CL1-CX3CR1 signaling in selective neuronal Atg7 KO mice on the mRNA level, especially in the cerebellum, and some *in vitro* and *in vivo* studies have shown that neurotoxic microglia activation in mice is suppressed by CX3CL1-CX3CR1 signaling.^51, 52, 53, 54^ These findings demonstrate that CX3CL1-CX3CR1 signaling might have a role in inhibiting microglia activation in juvenile mice with selective neuronal autophagy inhibition.

In conclusion, selective Atg7 deletion in neuronally committed cells prevents irradiation-induced caspase-3 activation, microglia activation, and inflammation and reduces irradiation-induced neural stem and progenitor cell death. Our results indicate that autophagy is a potential target for the prevention of irradiation-induced neural stem and progenitor cell death, especially when irradiation is used to treat malignant childhood brain tumors, including high-grade gliomas.

## Materials and methods

### Animals and ethical permission

Floxed *Atg7* mice were characterized as described previously^16, 18^ and were crossed with a nestin-Cre-driven line to produce Atg7^*flox/flox*; Nes-Cre^ KO (Atg7 KO) and Atg7^*flox/+*; Nes-Cre^ mice (WT). All of the mice were housed in a temperature-controlled and pathogen-free environment with a 12:12-h light–dark cycle. The genotyping of the pups was described previously.^18^ All experiments were approved by the animal research ethics committee (Gothenburg Committee of the Swedish Agricultural Agency) in accordance with national animal welfare legislation (114-2014).

### Irradiation procedure

Postnatal day 10 Atg7 KO and WT littermate pups of both sexes were anesthetized with a 50 mg/kg intraperitoneal injection of tribromoethanol (Avertin; Sigma-Aldrich, Stockholm, Sweden) and placed in a prone position (head to gantry) on a Styrofoam bed. The irradiation of the animals was performed using a linear accelerator (Varian Clinac 600CD; Radiation Oncology System LLC, San Diego, CA, USA) with 4 MV nominal photon energy and a dose rate of 2.3 Gy/min. The whole brain was irradiated with a single dose of 6 Gy to each mouse. The source-to-skin distance was ~99.5 cm. The head was covered with a 1 cm tissue-equivalent bolus material to obtain an even irradiation dose throughout the underlying tissue. After irradiation, the pups were returned to their dams and killed at 6 h after irradiation. The sham-irradiated controls were anesthetized but not irradiated.

### Immunohistochemistry staining

The mice were deeply anesthetized with 50 mg/ml phenobarbital and perfused intracardially with PBS and 5% buffered formaldehyde (Histofix; Histolab, Gothenburg, Sweden) at 6 h after irradiation. Brains were removed and fixed in 5% buffered formaldehyde at 4 °C for 24 h. After dehydration with graded ethanol and xylene, the brains were paraffin-embedded and cut into 5 *μ*m sagittal sections. Every 50th section in the hippocampus for active caspase-3, Ki-67, and Iba-1 staining was deparaffinized in xylene and rehydrated in graded ethanol concentrations. Antigen retrieval was performed by heating the sections in 10 mM boiling sodium citrate buffer (pH 6.0) for 10 min. Nonspecific binding was blocked for 30 min with 4% donkey or goat serum in PBS for 30 min. The primary antibodies were monoclonal rabbit antiactive caspase-3 (2.5 *μ*g/ml; BD Pharmingen, San Jose, CA, USA; 559565), rabbit anti-Ki-67 (2.5 *μ*g/ml; Abcam, Cambridge, UK; ab15580), and rabbit anti-Iba-1 (2.5 *μ*g/ml; Wako Pure Chemical Industries Ltd, Osaka, Japan; 019-19741). After incubating the sections with the primary antibodies overnight at 4 °C, the appropriate biotinylated secondary antibodies (1:200 dilution; all from Vector Laboratories, Burlingame, CA, USA) were added and incubated for 60 min at room temperature. After blocking endogenous peroxidase activity with 3% H_2_O_2_ for 10 min, the sections were visualized with Vectastain ABC Elite (Vector Laboratories) and 0.5 mg/ml 3,3,9-diaminobenzidine enhanced with ammonium nickel sulfate, *β*-d glucose, ammonium chloride and *β*-glucose oxidase. After dehydrating with graded ethanol and xylene, the sections were mounted using Vector mounting medium.

### Cell counting

The pyknotic cells, as indicated by HE staining, and the active caspase-3-, Ki-67-, and Iba-1-positive cells were counted in the SGZ of the dentate gyrus using stereology microscopy (MicroBrightField, Magdeburg, Germany) and expressed as the number of cells per mm. The counting area in the EGL, ML, cerebellar IGL, and cerebellar WM was traced in the cerebellum, and the number of cells was expressed as cells per mm^2^. The counting was performed by a person who did not have prior knowledge of the groups. Entire sets of sections from the hippocampal level were counted for all of the stainings with an interval of 250 *μ*m between sections. In the cerebellum, three sections were counted with an interval of 250 *μ*m between sections, and the second cerebellar lobule was selected for counting of HE, Ki-67, and active caspase-3 staining. Iba-1 was counted in the whole section. The Iba-1-positive cells were classified into ramified (surveillance phenotype/non-activated: characterized by long, ramified processes with comparatively small cell bodies), hyper-ramified (reactive/intermediate: characterized by thicker primary processes and retracting secondary processes), or unramified (activated), including bushy (characterized by swollen, truncated processes and enlarged cell bodies) or amoeboid (characterized by rounded macrophage-like morphology with no or few processes), microglia according to the morphological criteria.^55^

### RNA isolation and cDNA synthesis

Total RNA was isolated using the RNeasy Mini Kit (Qiagen, Hilden, Germany; 74104) according to the manufacturer's instructions. The concentration and purity of all RNA samples were determined using a Nanodrop spectrophotometer (Nanodrop Technologies, Wilmington, DE, USA). The integrity of RNA was measured using the Experion RNA StdSens Analysis Kit (Bio-Rad, Hercules, CA, USA; 7007103) on an Automated Electrophoresis Station (Bio-Rad, Hercules, CA, USA). One microgram of total RNA was reverse transcribed using the QuantiTect Reverse Transcription Kit (Qiagen; 205311).

### Quantitative real-time PCR

Quantitative real-time PCR was performed using the LightCycler 480 Instrument (Roche Diagnostics, Mannheim, Germany) and the SYBR Green technique according to the manufacturer's instructions. The primers used in the qRT-PCR reactions were designed by Beacon Designer software (free trial, PREMIER Biosoft, Palo Alto, CA, USA) and included the stem cell and proliferation genes *Ki-67* (sense: 5′-GCCTCCTAATACACCACTGA-3′ antisense: 5′-CCGTTCCTTGATGATTGTCTT-3′) and *SOX2* (sense: 5′-CGCAGACCTACATGAACG-3′ antisense: 5′-CTCGGACTTGACCACAGA-3′) and the CX3CL1-CX3CR1 signaling pathway genes *CX3CR1* (sense: 5′-GTCTGGTGGGAAATCTGTTG-3′ antisense: 5′-GGCTGATGAGGTAGTGAGT-3′) and *CX3CL1* (sense: 5′-CCTCAGAGCATTGGAAGTTT-3′ antisense: 5′-TTGAAGGTGAAGTAGTGGACA-3′). The reference gene was *sdha* (sense: 5′-TTGCCTTGCCAGGACTTA-3′ antisense: 5′-CACCTTGACTGTTGATGAGAAT-3′). The relative expression levels of mRNAs were calculated by the method of geometric averaging of multiple internal control genes.

### Multiplex cytokine/chemokine assay

Cytokines and chemokines were measured in the dentate gyrus and cerebellum homogenate supernatant fractions from postnatal day 10  WT and Atg7 KO mice at 6 h after irradiation. Samples were prepared according to the manufacturer's protocol. Protein concentration was measured with the BCA protein assay (Sigma, St. Louis, MO, USA; A2058). Levels of IL-1*β*, IL-2, IL-4, IL-6, IL-10, KC, and CCL2 were simultaneously measured using the Luminex Multiplex Cytokine Assay (Merck Chemicals and Life Science AB, Billerica, MA, USA). The results were normalized to the amount of protein in the sample.

### Statistical analysis

The Statistical Package for the Social Sciences 17.0 (SPSS; IBM, New York, NY, USA) was used for all analyses. Comparisons between groups were performed by Student's *t*-test, and data with unequal variance were compared with the Mann–Whitney *U*-test. Two-way ANOVA followed by a Bonferroni *post hoc* test was used for multiple comparison correction of data from more than two groups. Results are presented as means±S.E.M., and *P*<0.05 was considered statistically significant.

## References

[bib1] Benadiba J, Michel G, Auquier P, Chastagner P, Kanold J, Poiree M et al. Health status and quality of life of long-term survivors of childhood acute leukemia: the impact of central nervous system irradiation. J Pediatr Hematol Oncol 2015; 37: 109–116. 24936742 10.1097/MPH.0000000000000209

[bib2] Yock TI, Yeap BY, Ebb DH, Weyman E, Eaton BR, Sherry NA et al. Long-term toxic effects of proton radiotherapy for paediatric medulloblastoma: a phase 2 single-arm study. Lancet Oncol 2016; 17: 287–298. 26830377 10.1016/S1470-2045(15)00167-9

[bib3] Zhu C, Huang Z, Gao J, Zhang Y, Wang X, Karlsson N et al. Irradiation to the immature brain attenuates neurogenesis and exacerbates subsequent hypoxic-ischemic brain injury in the adult. J Neurochem 2009; 111: 1447–1456. 19799713 10.1111/j.1471-4159.2009.06413.x

[bib4] Kahalley LS, Ris MD, Grosshans DR, Okcu MF, Paulino AC, Chintagumpala M et al. Comparing intelligence quotient change after treatment with proton versus photon radiation therapy for pediatric brain tumors. J Clin Oncol 2016; 34: 1043–1049. 26811522 10.1200/JCO.2015.62.1383 PMC4872015

[bib5] Blomstrand M, Kalm M, Grander R, Bjork-Eriksson T, Blomgren K. Different reactions to irradiation in the juvenile and adult hippocampus. Int J Radiat Biol 2014; 90: 807–815. 25004947 10.3109/09553002.2014.942015

[bib6] Duffner PK. Risk factors for cognitive decline in children treated for brain tumors. Eur J Paediatr Neurol 2010; 14: 106–115. 19931477 10.1016/j.ejpn.2009.10.005

[bib7] Fukuda A, Fukuda H, Swanpalmer J, Hertzman S, Lannering B, Marky I et al. Age-dependent sensitivity of the developing brain to irradiation is correlated with the number and vulnerability of progenitor cells. J Neurochem 2005; 92: 569–584. 15659227 10.1111/j.1471-4159.2004.02894.x

[bib8] Laprie A, Hu Y, Alapetite C, Carrie C, Habrand JL, Bolle S et al. Paediatric brain tumours: a review of radiotherapy, state of the art and challenges for the future regarding protontherapy and carbontherapy. Cancer Radiother 2015; 19: 775–789. 26548600 10.1016/j.canrad.2015.05.028

[bib9] Bostrom M, Kalm M, Karlsson N, Hellstrom Erkenstam N, Blomgren K. Irradiation to the young mouse brain caused long-term, progressive depletion of neurogenesis but did not disrupt the neurovascular niche. J Cereb Blood Flow Metab 2013; 33: 935–943. 23486289 10.1038/jcbfm.2013.34 PMC3677115

[bib10] Kalm M, Karlsson N, Nilsson MK, Blomgren K. Loss of hippocampal neurogenesis, increased novelty-induced activity, decreased home cage activity, and impaired reversal learning one year after irradiation of the young mouse brain. Exp Neurol 2013; 247: 402–409. 23333566 10.1016/j.expneurol.2013.01.006

[bib11] Huo K, Sun Y, Li H, Du X, Wang X, Karlsson N et al. Lithium reduced neural progenitor apoptosis in the hippocampus and ameliorated functional deficits after irradiation to the immature mouse brain. Mol Cell Neurosci 2012; 51: 32–42. 22800605 10.1016/j.mcn.2012.07.002

[bib12] Fukuda H, Fukuda A, Zhu C, Korhonen L, Swanpalmer J, Hertzman S et al. Irradiation-induced progenitor cell death in the developing brain is resistant to erythropoietin treatment and caspase inhibition. Cell Death Differ 2004; 11: 1166–1178. 15243583 10.1038/sj.cdd.4401472

[bib13] Zhu C, Xu F, Fukuda A, Wang X, Fukuda H, Korhonen L et al. X chromosome-linked inhibitor of apoptosis protein reduces oxidative stress after cerebral irradiation or hypoxia–ischemia through up-regulation of mitochondrial antioxidants. Eur J Neurosci 2007; 26: 3402–3410. 18052985 10.1111/j.1460-9568.2007.05948.x

[bib14] Osato K, Sato Y, Ochiishi T, Osato A, Zhu C, Sato M et al. Apoptosis-inducing factor deficiency decreases the proliferation rate and protects the subventricular zone against ionizing radiation. Cell Death Dis 2010; 1: e84. 21368857 10.1038/cddis.2010.63 PMC3035904

[bib15] Oppenheim RW, Blomgren K, Ethell DW, Koike M, Komatsu M, Prevette D et al. Developing postmitotic mammalian neurons *in vivo* lacking Apaf-1 undergo programmed cell death by a caspase-independent, nonapoptotic pathway involving autophagy. J Neurosci 2008; 28: 1490–1497. 18256270 10.1523/JNEUROSCI.4575-07.2008 PMC6671586

[bib16] Komatsu M, Waguri S, Chiba T, Murata S, Iwata J, Tanida I et al. Loss of autophagy in the central nervous system causes neurodegeneration in mice. Nature 2006; 441: 880–884. 16625205 10.1038/nature04723

[bib17] Puyal J, Ginet V, Clarke PG. Multiple interacting cell death mechanisms in the mediation of excitotoxicity and ischemic brain damage: a challenge for neuroprotection. Prog Neurobiol 2013; 105: 24–48. 23567504 10.1016/j.pneurobio.2013.03.002

[bib18] Xie C, Ginet V, Sun Y, Koike M, Zhou K, Li T et al. Neuroprotection by selective neuronal deletion of Atg7 in neonatal brain injury. Autophagy 2016; 12: 410–423. 26727396 10.1080/15548627.2015.1132134 PMC4835980

[bib19] Liu C, He W, Jin M, Li H, Xu H, Liu H et al. Blockage of autophagy in C6 glioma cells enhanced radiosensitivity possibly by attenuating DNA-PK-dependent DSB due to limited Ku nuclear translocation and DNA binding. Curr Mol Med 2015; 15: 663–673. 26321753 10.2174/1566524015666150831141112

[bib20] Kalm M, Fukuda A, Fukuda H, Ohrfelt A, Lannering B, Bjork-Eriksson T et al. Transient inflammation in neurogenic regions after irradiation of the developing brain. Radiat Res 2009; 171: 66–76. 19138045 10.1667/RR1269.1

[bib21] Han W, Umekawa T, Zhou K, Zhang XM, Ohshima M, Dominguez CA et al. Cranial irradiation induces transient microglia accumulation, followed by long-lasting inflammation and loss of microglia. Oncotarget 2016; 7: 82305–82323. 27793054 10.18632/oncotarget.12929 PMC5347693

[bib22] Monje ML, Toda H, Palmer TD. Inflammatory blockade restores adult hippocampal neurogenesis. Science 2003; 302: 1760–1765. 14615545 10.1126/science.1088417

[bib23] Koike M, Shibata M, Tadakoshi M, Gotoh K, Komatsu M, Waguri S et al. Inhibition of autophagy prevents hippocampal pyramidal neuron death after hypoxic-ischemic injury. Am J Pathol 2008; 172: 454–469. 18187572 10.2353/ajpath.2008.070876 PMC2312361

[bib24] Komatsu M, Waguri S, Ueno T, Iwata J, Murata S, Tanida I et al. Impairment of starvation-induced and constitutive autophagy in Atg7-deficient mice. J Cell Biol 2005; 169: 425–434. 15866887 10.1083/jcb.200412022 PMC2171928

[bib25] Button RW, Luo S, Rubinsztein DC. Autophagic activity in neuronal cell death. Neurosci Bull 2015; 31: 382–394. 26077705 10.1007/s12264-015-1528-y PMC5563708

[bib26] Kanamori H, Takemura G, Maruyama R, Goto K, Tsujimoto A, Ogino A et al. Functional significance and morphological characterization of starvation-induced autophagy in the adult heart. Am J Pathol 2009; 174: 1705–1714. 19342365 10.2353/ajpath.2009.080875 PMC2671259

[bib27] Carloni S, Girelli S, Scopa C, Buonocore G, Longini M, Balduini W. Activation of autophagy and Akt/CREB signaling play an equivalent role in the neuroprotective effect of rapamycin in neonatal hypoxia-ischemia. Autophagy 2010; 6: 366–377. 20168088 10.4161/auto.6.3.11261

[bib28] Ginet V, Pittet MP, Rummel C, Osterheld MC, Meuli R, Clarke PG et al. Dying neurons in thalamus of asphyxiated term newborns and rats are autophagic. Ann Neurol 2014; 76: 695–711. 25146903 10.1002/ana.24257

[bib29] Zhou ZR, Zhu XD, Zhao W, Qu S, Su F, Huang ST et al. Poly(ADP-ribose) polymerase-1 regulates the mechanism of irradiation-induced CNE-2 human nasopharyngeal carcinoma cell autophagy and inhibition of autophagy contributes to the radiation sensitization of CNE-2 cells. Oncol Rep 2013; 29: 2498–2506. 23563481 10.3892/or.2013.2382

[bib30] Ito H, Daido S, Kanzawa T, Kondo S, Kondo Y. Radiation-induced autophagy is associated with LC3 and its inhibition sensitizes malignant glioma cells. Int J Oncol 2005; 26: 1401–1410. 15809734

[bib31] Ye H, Chen M, Cao F, Huang H, Zhan R, Zheng X. Chloroquine, an autophagy inhibitor, potentiates the radiosensitivity of glioma initiating cells by inhibiting autophagy and activating apoptosis. BMC Neurol 2016; 16: 178. 27644442 10.1186/s12883-016-0700-6 PMC5029068

[bib32] Balentova S, Adamkov M. Molecular, cellular and functional effects of radiation-induced brain injury: a review. Int J Mol Sci 2015; 16: 27796–27815. 26610477 10.3390/ijms161126068 PMC4661926

[bib33] Maiuri MC, Zalckvar E, Kimchi A, Kroemer G. Self-eating and self-killing: crosstalk between autophagy and apoptosis. Nat Rev Mol Cell Biol 2007; 8: 741–752. 17717517 10.1038/nrm2239

[bib34] Wu HJ, Pu JL, Krafft PR, Zhang JM, Chen S. The molecular mechanisms between autophagy and apoptosis: potential role in central nervous system disorders. Cell Mol Neurobiol 2015; 35: 85–99. 25257832 10.1007/s10571-014-0116-z PMC11488065

[bib35] Galluzzi L, Bravo-San Pedro JM, Blomgren K, Kroemer G. Autophagy in acute brain injury. Nat Rev Neurosci 2016; 17: 467–484. 27256553 10.1038/nrn.2016.51

[bib36] Lv X, Jiang H, Li B, Liang Q, Wang S, Zhao Q et al. The crucial role of Atg5 in cortical neurogenesis during early brain development. Sci Rep 2014; 4: 6010. 25109817 10.1038/srep06010 PMC4127499

[bib37] Hellstrom NA, Bjork-Eriksson T, Blomgren K, Kuhn HG. Differential recovery of neural stem cells in the subventricular zone and dentate gyrus after ionizing radiation. Stem cells 2009; 27: 634–641. 19056908 10.1634/stemcells.2008-0732

[bib38] Hailer NP, Grampp A, Nitsch R. Proliferation of microglia and astrocytes in the dentate gyrus following entorhinal cortex lesion: a quantitative bromodeoxyuridine-labelling study. Eur J Neurosci 1999; 11: 3359–3364. 10510203 10.1046/j.1460-9568.1999.00808.x

[bib39] Lewis CA, Manning J, Rossi F, Krieger C. The neuroinflammatory response in ALS: the roles of microglia and T Cclls. Neurol Res Int 2012; 2012: 803701. 22666587 10.1155/2012/803701 PMC3362167

[bib40] Hwang SY, Jung JS, Kim TH, Lim SJ, Oh ES, Kim JY et al. Ionizing radiation induces astrocyte gliosis through microglia activation. Neurobiol Dis 2006; 21: 457–467. 16202616 10.1016/j.nbd.2005.08.006

[bib41] Levesque SA, Pare A, Mailhot B, Bellver-Landete V, Kebir H, Lecuyer MA et al. Myeloid cell transmigration across the CNS vasculature triggers IL-1beta-driven neuroinflammation during autoimmune encephalomyelitis in mice. J Exp Med 2016; 213: 929–949. 27139491 10.1084/jem.20151437 PMC4886360

[bib42] Lee WH, Sonntag WE, Mitschelen M, Yan H, Lee YW. Irradiation induces regionally specific alterations in pro-inflammatory environments in rat brain. Int J Radiat Biol 2010; 86: 132–144. 20148699 10.3109/09553000903419346 PMC2827151

[bib43] Li H, Li Q, Du X, Sun Y, Wang X, Kroemer G et al. Lithium-mediated long-term neuroprotection in neonatal rat hypoxia-ischemia is associated with antiinflammatory effects and enhanced proliferation and survival of neural stem/progenitor cells. J Cereb Blood Flow Metab 2011; 31: 2106–2115. 21587270 10.1038/jcbfm.2011.75 PMC3208156

[bib44] Yuan B, Shen H, Lin L, Su T, Zhong L, Yang Z. Autophagy Promotes Microglia Activation Through Beclin-1-Atg5 Pathway in Intracerebral Hemorrhage. Mol Neurobiol 2016; 54: 115–124. 26732594 10.1007/s12035-015-9642-z

[bib45] Su P, Zhang J, Wang D, Zhao F, Cao Z, Aschner M et al. The role of autophagy in modulation of neuroinflammation in microglia. Neuroscience 2016; 319: 155–167. 26827945 10.1016/j.neuroscience.2016.01.035

[bib46] Levine B, Mizushima N, Virgin HW. Autophagy in immunity and inflammation. Nature 2011; 469: 323–335. 21248839 10.1038/nature09782 PMC3131688

[bib47] Limatola C, Ransohoff RM. Modulating neurotoxicity through CX3CL1/CX3CR1 signaling. Front Cell Neurosci 2014; 8: 229. 25152714 10.3389/fncel.2014.00229 PMC4126442

[bib48] Cipriani R, Villa P, Chece G, Lauro C, Paladini A, Micotti E et al. CX3CL1 is neuroprotective in permanent focal cerebral ischemia in rodents. J Neurosci 2011; 31: 16327–16335. 22072684 10.1523/JNEUROSCI.3611-11.2011 PMC6633249

[bib49] Tang Z, Gan Y, Liu Q, Yin JX, Liu Q, Shi J et al. CX3CR1 deficiency suppresses activation and neurotoxicity of microglia/macrophage in experimental ischemic stroke. J Neuroinflamm 2014; 11: 26. 10.1186/1742-2094-11-26 PMC3942808 24490760

[bib50] Liu Y, Wu XM, Luo QQ, Huang S, Yang QW, Wang FX et al. CX3CL1/CX3CR1-mediated microglia activation plays a detrimental role in ischemic mice brain via p38MAPK/PKC pathway. J Cereb Blood Flow Metab 2015; 35: 1623–1631. 25966946 10.1038/jcbfm.2015.97 PMC4640309

[bib51] Biber K, de Jong EK, van Weering HR, Boddeke HW. Chemokines and their receptors in central nervous system disease. Curr Drug Targets 2006; 7: 29–46. 16454698 10.2174/138945006775270196

[bib52] Zujovic V, Benavides J, Vige X, Carter C, Taupin V. Fractalkine modulates TNF-alpha secretion and neurotoxicity induced by microglial activation. Glia 2000; 29: 305–315. 10652441

[bib53] Mizuno T, Kawanokuchi J, Numata K, Suzumura A. Production and neuroprotective functions of fractalkine in the central nervous system. Brain Res 2003; 979: 65–70. 12850572 10.1016/s0006-8993(03)02867-1

[bib54] Cardona AE, Pioro EP, Sasse ME, Kostenko V, Cardona SM, Dijkstra IM et al. Control of microglial neurotoxicity by the fractalkine receptor. Nat Neurosci 2006; 9: 917–924. 16732273 10.1038/nn1715

[bib55] Karperien A, Ahammer H, Jelinek HF. Quantitating the subtleties of microglial morphology with fractal analysis. Front Cell Neurosci 2013; 7: 3. 23386810 10.3389/fncel.2013.00003 PMC3558688

